# Inhibitor-Induced Conformational Stabilization and Structural Alteration of a Mip-Like Peptidyl Prolyl *cis-trans* Isomerase and Its C-Terminal Domain

**DOI:** 10.1371/journal.pone.0102891

**Published:** 2014-07-29

**Authors:** Soumitra Polley, Biswanath Jana, Gopal Chakrabarti, Subrata Sau

**Affiliations:** 1 Department of Biochemistry, Bose Institute, Kolkata, West Bengal, India; 2 Dr. B.C. Guha Centre for Genetic Engineering & Biotechnology, University of Calcutta, Kolkata, West Bengal, India; CNR, Italy

## Abstract

FKBP22, an *Escherichia coli*-encoded PPIase (peptidyl-prolyl *cis*-*trans* isomerase) enzyme, shares substantial identity with the Mip-like pathogenic factors, caries two domains, exists as a dimer in solution and binds some immunosuppressive drugs (such as FK506 and rapamycin) using its C-terminal domain (CTD). To understand the effects of these drugs on the structure and stability of the Mip-like proteins, rFKBP22 (a chimeric FKBP22) and CTD^+^ (a CTD variant) have been studied in the presence and absence of rapamycin using different probes. We demonstrated that rapamycin binding causes minor structural alterations of rFKBP22 and CTD^+^. Both the proteins (equilibrated with rapamycin) were unfolded via the formation of intermediates in the presence of urea. Further study revealed that thermal unfolding of both rFKBP22 and rapamycin-saturated rFKBP22 occurred by a three-state mechanism with the synthesis of intermediates. Intermediate from the rapamycin-equilibrated rFKBP22 was formed at a comparatively higher temperature. All intermediates carried substantial extents of secondary and tertiary structures. Intermediate resulted from the thermal unfolding of rFKBP22 existed as the dimers in solution, carried an increased extent of hydrophobic surface and possessed relatively higher rapamycin binding activity. Despite the formation of intermediates, both the thermal and urea-induced unfolding reactions were reversible in nature. Unfolding studies also indicated the considerable stabilization of both proteins by rapamycin binding. The data suggest that rFKBP22 or CTD^+^ could be exploited to screen the rapamycin-like inhibitors in the future.

## Introduction

FKBP22, a protein-folding catalyst expressed by *Escherichia coli* [1], harbors 206 amino acid residues, forms dimers in solution and shares significant identity with *Shewanella sp*. FKBP22 [2], *Salmonella typhimurium* FkpA [3], and the Mip (macrophage infectivity potentiator)-like virulence proteins from several human and plant pathogens (namely, *Legionella pneumophilla*, *Chlamydia trachomatis*, *Trypanosoma cruzi*, *Neisseria gonorrhoeae*, *Xanthomonas campestris*, etc.) [4]–[10]. Rapamycin and FK506 but not the juglone or cyclosporin A inhibited the PPIase (peptidyl-prolyl *cis-trans* isomerase; EC 5.1.2.8) activity of the Mip proteins and their orthologs [11]. Structural investigations suggested a V-shaped structure for the dimeric Mip-like proteins [12]–[15]. Each dumbbell-shaped monomer is composed of a C-terminal domain (CTD), a hinge region, and an N-terminal domain (NTD). While the N-terminal domain is responsible for dimerization of the molecule, the C-terminal domain possesses the substrate and the inhibitor binding sites. Conversely, the ‘hinge’ region that constitutes the branches of the V-shaped conformation and connects the two domains is composed of a protease-sensitive α-helix with the length of ∼6.5 nm [13], [14]. The V-shaped structure including the hinge region was reported to be critical for the PPIase activity of Mip-like proteins with a protein substrate [15]–[17]. A recent study suggested that GdnCl- and urea-induced denaturation of a chimeric *E. coli* FKBP22 follow a three-state and a two-state mechanism, respectively. Remarkably, intermediates produced during the denaturation of a recombinant FKBP22 with GdnCl were not molten globules but believed to be made of different incompletely denatured multimers of this protein [12].

The tertiary structure of a biologically active protein is typically stabilized by various non-covalent bonds (such as ionic bonds, van der Waal interactions, hydrogen bonds, and hydrophobic interactions) and sometimes by disulfide bonds. Of the stabilizing factors, hydrophobic interaction is the key contributor towards the stability as well as the folding of a protein in an aqueous environment [18]–[23]. The folding usually pushes the hydrophobic side chains of non-polar amino acids in a linear polypeptide into the interior of its three-dimensional form. Binding of the ligands to proteins not only alters their hydrophobic interactions but also stabilizes them along with increasing of their midpoints of thermal or chemical denaturation [23]–[28]. Sometimes ligand binding also causes substantial conformational alteration of proteins. Binding of FK506 or rapamycin to human FKBP12 caused the burial of several surface-accessible nonpolar amino acid residues at the drug binding site [29]–[31] and augmented its stability [27], [28]. Binding of FK506 to the *E. coli* FkpA also buried a surface area of 380 Å^2^ at its protein-drug interface [14]. The C-terminal domain of *Legionella* Mip revealed a little conformational rearrangement in the presence of rapamycin [32].

CTD^+^, the C-terminal domain of *E. coli* FKBP22 with a truncated hinge, shares significant identity with human FKBP12 [27] and the C-terminal domains of many Mip-like proteins [2]–[10]. The tertiary structure of isolated CTD^+^ appears to be a little different than that of the C-terminal domain in FKBP22 [12]. This domain was also reported to be less stable than both rFKBP22 and NTD^+^ (NTD of *E. coli* FKBP22 with a long hinge region). Unlike FKBP22, GdnCl-induced denaturation of CTD^+^ followed a two-state mechanism [12]. Despite the altered structure, CTD^+^ bound rapamycin nearly similarly to that of a recombinant FKBP22 [12]. Proteins, which are orthologous to CTD^+^, exhibited PPIase activity with the peptide substrates as well [16], [29], [32]. To date, very little is known about the folding - unfolding mechanisms, structures, and the stabilities of the Mip-like proteins and their C-terminal domains in the presence of rapamycin or FK506. Under the context of emergence and dissemination of the antimicrobial-resistant strains of the Mip-producing human pathogens, stability data of a Mip protein (or its C-terminal domain) in the presence and absence of a cognate drug may provide a solid foundation in screening new drugs capable of killing these pathogens [28], [32], [33]. Using rFKBP22 (a recombinant *E. coli* FKBP22) [17] and CTD^+^ as the model proteins, we have demonstrated that rapamycin binding causes minor structural alterations in these proteins. Urea and temperature appeared to unfold these proteins (pre-equilibrated with rapamycin) via the synthesis of intermediates. Both CTD^+^ and rFKBP22 were stabilized substantially in the presence of rapamycin. Additional investigation revealed that thermal unfolding of rFKBP22 (bound with or without rapamycin) occurs via the formation of an active dimeric intermediate.

## Materials and Methods

### Materials

Acrylamide, 8-anilino-1-napthalenesulfonate (ANS), bis-acrylamide, glutaraldehyde, IPTG (isopropyl β-D-1-thiogalactopyranoside), PMSF (phenylmethane sulfonylfluoride), FK506 and urea were bought from SRL, Merck or Sigma. All other reagents were of the maximum purity available. The Ni-NTA resin, rapamycin, markers (DNA and protein), and RNase T1 were purchased from Qiagen, BioVision, Genetix Biotech Asia Pvt Ltd. and Fermentas, respectively. The anti-His antibody and goat anti-mouse antibody tagged with alkaline phosphatase (IgG1-AP) were purchased from Santa Cruz Biotechnology Inc. rFKBP22, CTD^+^, and NTD^+^ were purified as described previously [12], [17].

### Bioinformatic analyses

Sequence similarity search, alignment of amino acid sequences, and recognition of protease cleavage site in the proteins were performed by blastp (http://www.ncbi.nlm.nih.gov/BLAST), ClustalW (http://www.ebi.ac.uk/clustalw), and PeptideCutter (http://www.expasy.org) program, respectively.

The three-dimensional model structure of CTD^+^ - rapamycin complex was built by EasyModeller server [34] using the C-terminal domain-specific sequence (amino acid residues 83–206) of *E. coli* FKBP22 and the NMR structure (Protein Data Bank entry 2vcd) of *Legionella* Mip-specific C-terminal domain [32], which is bound with rapamycin. Similarly, a model structure of CTD^+^ was also developed using the NMR structure (Protein Data Bank entry 2uz5) of C-terminal domain of *Legionella* Mip [32] and the above sequence of *E. coli* FKBP22. Superimposition of the two model structures was carried out by Swiss-Pdb Viewer (http://ExPasy.org).

### Basic analysis of protein

The total protein content was determined by a standard procedure as reported earlier [12], [35]. SDS-PAGE, staining of the polyacrylamide gel and Western blotting experiment were carried out as described [36], [37]. Glutaraldehyde-mediated chemical crosslinking and limited proteolysis were performed essentially as described before [12]. The molar concentration of rFKBP22, CTD^+^, and NTD^+^ were estimated using the molecular mass of their relevant monomers. Considering that one rapamycin molecule binds to each rFKBP22 monomer (as evident from our modeling study), the equilibrium dissociation constant (*K*_d_) of rapamycin - rFKBP22 interaction was determined essentially by a procedure as reported earlier [12].

### Structural and unfolding probes

The far-UV Circular Dichroism (CD) spectrum (200–260 nm), intrinsic Trp fluorescence spectrum (λ_ex_ = 295 nm and λ_em_ = 300–400 nm), and ANS fluorescence spectrum (λ_ex_ = 360 nm and λ_em_ = 480 nm) of different proteins were recorded by the standard procedures [12], [38], [39]. Thermal aggregation of a protein was observed by determining its light scattering at 360 nm [38]. To make the protein-rapamycin complex, each protein (5–10 µM) in buffer B [12] was incubated with rapamycin (10–20 µM) for 20–25 min at 4°C prior to the collection of its spectroscopic data. Unless otherwise stated, the ratio of protein to rapamycin concentrations were kept identical (as above) in all other experiments described here.

To study the chemical-induced unfolding of rapamycin (or FK506)-saturated/unsaturated proteins, they were incubated with 0–7 M urea individually for 16–18 h at 4°C prior to the recording of their far-UV CD and the intrinsic tryptophan fluorescence spectra. Unlike rapamycin, four folds molar excess of FK506 was used in the study. Refolding of the urea-denatured protein was monitored by tryptophan fluorescence spectroscopy [12]. Unfolding and refolding of 12.5 µM protein (saturated/unsaturated with 25 µM rapamycin) were also investigated using transverse urea gradient gel electrophoresis [12], [40].

To study the thermal unfolding of the rapamycin-equilibrated/unequilibrated proteins, their far-UV CD and the intrinsic tryptophan fluorescence spectra were recorded at 25°–85°C. At each temperature, samples were incubated for 3–5 min prior to the collection of spectra.

To study the reversibility of thermal unfolding, temperature of rFKBP22 (pre-bound or unbound with rapamycin) in buffer B [12] was raised gradually from 25° to 85°C (at the rate of 1°C per min) followed by the reduction of its temperature slowly (at the same rate) to 25°C. Similarly, buffer B (containing rapamycin or no rapamycin) was treated. The far-UV CD and the tryptophan fluorescence spectra of all cooled samples and buffer were recorded essentially as described above. The spectroscopic signal of the cooled buffer was deducted from that of the refolded protein. Far-UV CD and the tryptophan fluorescence spectra of both the native and unfolded rFKBP22 were also recorded simultaneously for comparison. Samples carrying equimolar concentrations of rFKBP22 were used in all experiments. The two-domain structure and the catalytic activity of the refolded rFKBP22 were also checked by the partial trypsinolysis and by the RNaseT1 refolding assay, respectively [12].

### Determination of thermodynamic parameters of unfolding

Urea-induced unfolding of proteins (pre-equilibrated with or without rapamycin) apparently followed a two-state mechanism (see below) [41]. To corroborate the above suggestions, the fraction of denatured protein molecules (*F*_U_), resulting from different spectroscopic probes (Figure S4), was determined as described [12], [41]. By globally fitting the urea-induced unfolding data to the standard equations [12], [41], Δ*G* ^W^ (free energy change at 0 M urea), *m* (cooperativity parameter of urea-induced unfolding), *C*_m_ (urea concentration at the midpoint of unfolding transition, i.e. urea concentration at which Δ*G* = 0), and ΔΔ*G* (the difference of free energy change between the rapamycin-bound protein and rapamycin-unbound protein) were determined.

The thermal unfolding of rFKBP22 (pre-equilibrated with or without rapamycin) seems to follow a three-state mechanism (see below) [41], [42]. By globally fitting the thermal unfolding data to the standard equations [42], thermodynamic parameters (*T*_m1_, Δ*H*_m1_ and ΔΔ*G*1) for the native to intermediate, and those (*T*_m2_, Δ*H*_m2_ and ΔΔ*G*2) for the intermediate to unfolded state transition were determined. *T*_m_, Δ*H*_m_ and ΔΔ*G* indicate the melting temperature, enthalpy change of the rapamycin-unbound protein at its *T*_m_, and difference in the free-energy change between the rapamycin-unbound protein and rapamycin-bound protein, respectively.

To determine the temperature (also represented as *T*_m_) at which 50% of the proteins (bound or unbound with rapamycin) were digested by thermolysin, the band intensities of all undigested proteins were determined from the gel images (see below), normalized with respect to those from the same proteins at 30°C, and plotted against the corresponding temperatures.

### Statistical Analysis

We have presented all results as the means of at least three separate experiments with standard deviation. Standard deviation, mean, and *p* values were calculated using specific functions from Microsoft Excel. The two results were judged significant when the associated *p* value was less than 0.05.

## Results and Discussion

### Effect of Rapamycin on the Structures of rFKBP22 and CTD^+^

**Figure 1:**
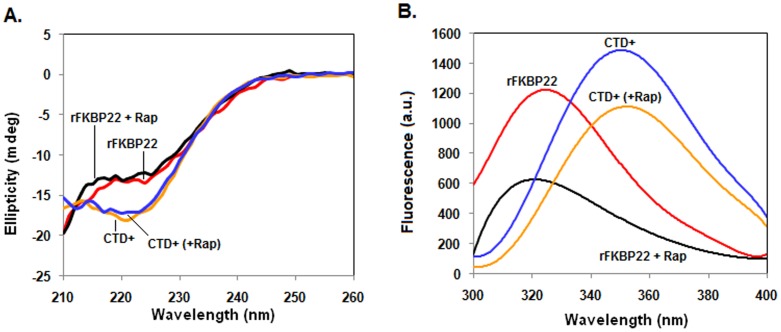
Far-UV CD (A) and intrinsic Trp fluorescence (B) spectra of the designated proteins (bound with or without rapamycin) were recorded at room temperature.

Ligand binding to a protein usually changes its structure and stability. To understand whether rapamycin binding causes any structural alteration of the C-terminal domain of *E. coli* FKBP22, we developed the model structures of both CTD^+^ and CTD^+^-rapamycin complex using standard procedures (See Materials and methods for details). The model structure of CTD^+^-rapamycin (Figure S1A) did not look identical to that of CTD^+^ (Figure S1B). The structure of CTD^+^ when superimposed to that of its rapamycin-bound form, however, yielded a root mean square deviation of 2.74 Å (Figure S1C), indicating that there is a minor structural variation of CTD^+^ in the presence of rapamycin. To verify whether rapamycin binding affects the secondary and tertiary structures of CTD^+^, the far-UV CD and the intrinsic Trp fluorescence spectra of this protein was recorded in the presence and absence of rapamycin. Figure 1A shows that the spectrum of rapamycin-bound CTD^+^ did not completely overlap with that of rapamycin-unbound CTD^+^. The far-UV CD spectrum of rFKBP22 was also not fully identical to that of the rapamycin-bound rFKBP22 (Figure 1A). Unlike the CD spectra, the Trp fluorescence spectrum of rapamycin-bound CTD^+^/rFKBP22 is completely different from that of rapamycin-unbound CTD^+^/rFKBP22 (Figure 1B). There is a considerable reduction of the fluorescence intensities of both rFKBP22 and CTD^+^ in the presence of two folds molar excess of rapamycin. The emission maxima of the spectra of two proteins were altered but in different way in the presence of rapamycin. While the spectrum of rapamycin-saturated rFKBP22 was associated with a 5 nm blue-shifted emission maxima, that of rapamycin-bound CTD^+^ was coupled with a 2 nm red-shifted emission maxima. Taken together, rapamycin binding altered both the secondary and tertiary structures of both CTD^+^ and rFKBP22 to some extent. Binding of FK506 to rFKBP22 also appeared to alter its conformation to some extent (Figure S2).

The drug-induced (small) conformational change that we noticed in rFKBP22 and CTD^+^ was also noted in some FKBP22 orthologs in the presence of cognate drugs [11], [13], [14], [29]–[32]. More precisely, the regions, formed by the amino acid residues Thr144 to Pro150 and Val179 to Thr199 in *Legionella* Mip, displayed a little conformational rearrangement upon its binding to rapamycin [32]. *E. coli* FKBP22 appeared to possess two regions that share moderate homology with the above two regions of *Legionella* Mip (Figure S3). It is not clear now whether drug binding caused conformational rearrangement in these two homologous regions of CTD^+^ or rFKBP22.

### Urea-induced unfolding of the rapamycin-bound/unbound proteins

**Figure 2:**
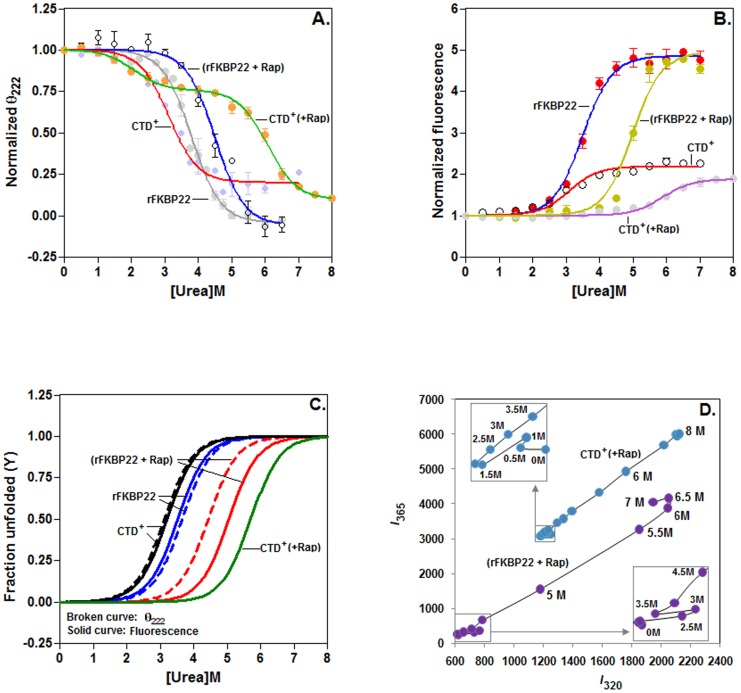
Unfolding of rFKBP22 and CTD^+^ (bound or unbound with rapamycin) by urea. (A) The θ_222_ (ellipticity at 222 nm) values, extracted from the CD spectra (Figures S4A–4D) of the indicated proteins (saturated with or without rapamycin) at 0–7/8 M urea, were normalized and plotted as described [12]. (B) The Trp fluorescence intensities at 330 nm (for rFKBP22 or rapamycin-bound rFKBP22) and at 347 nm (for CTD^+^ or rapamycin-saturated CTD^+^) were similarly derived from the fluorescence spectra (Figures S4E-4H), normalized and plotted against the matching urea concentrations. All lines through the fluorescence or ellipticity values represent the best-fit curves. (C) Mechanism of urea-induced unfolding. Fraction of denatured protein molecules (*Y*) in the absence and presence of rapamycin were determined from the θ_222_ or Trp fluorescence intensity values (collected as stated above) using equation 2 and plotted against the relevant urea concentrations values as explained in Materials and methods. (D) Phase diagrams show the denaturation of rapamycin-equilibrated rFKBP22 or CTD^+^ at 0–7/8 M urea. *I*_320_ and *I*_365_ indicate the fluorescence intensity values (obtained from Figures S4E–4H) of the indicated proteins at 320 nm and at 365 nm, respectively. Inset boxes show the enlarged view of the fluorescence data at 0–3.5/4.5 M urea.

**Figure 3:**
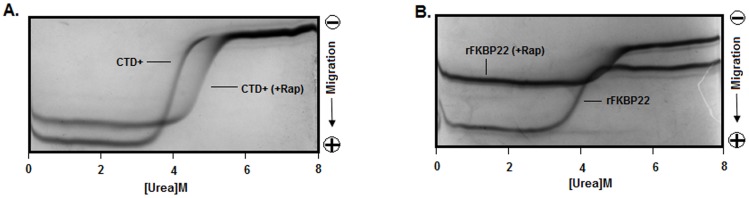
Mobility of the indicated native (A–B) proteins across the transverse urea gradient polyacrylamide gel.

To determine whether the unfolding mechanism of rapamycin-bound rFKBP22/CTD^+^ differs from that of rapamycin-unbound rFKBP22/CTD^+^ [12], we recorded the far-UV CD and the intrinsic Trp fluorescence spectra of all these proteins (pre-equilibrated with/without rapamycin) separately in the presence of 0–7/8 M urea (Figure S4 in the Supporting data). The curves, generated using the CD data of rapamycin-bound CTD^+^ and other proteins (rapamycin-saturated rFKBP22, rFKBP22, and CTD^+^), are apparently biphasic and monophasic, respectively (Figure 2A). In contrast, all of the protein-specific curves, produced using the Trp fluorescence intensity values, were observed to be monophasic at 0–7/8 M urea (Figure 2B). The *λ*_max_ (wavelength of emission maxima) values of the CTD^+^ (bound or unbound with rapamycin) remained static at 350 nm at 0–8 M urea, whereas, that of rFKBP22 (saturated or unsaturated with rapamycin) started increasing upon increasing the fluorescence intensity and reached to ∼349 nm when the fluorescence intensity values tended to saturate (Figure S4). Additional analyses of the above proteins (saturated/unsaturated with rapamycin) by the transverse urea gradient gel electrophoresis (TUGE) [17], [40] revealed that passage of all macromolecules across the 0–8 M urea gradient formed roughly the monophasic curve-shaped bands (Figure 3). The pre-transition, transition, and post-transition regions of no curve or monophasic curve-shaped bands overlapped with others, indicating that the initiation and completion of unfolding of each protein occurred at distinct urea concentration. The data (particularly those resulted from the Trp fluorescence spectroscopy and TUGE) also suggest that unfolding of rapamycin-equilibrated rFKBP22 or CTD^+^ was initiated and ended at significantly higher urea concentrations in comparison with that of rFKBP22 or CTD^+^ alone.

To determine whether the urea-induced unfolding of rapamycin-bound/unbound proteins (rFKBP22 and CTD^+^) were reversible in nature, these macromolecules were unfolded followed by their analyses with TUGE. The TUGE images showed that the denatured proteins (rFKBP22 and CTD^+^) in the presence/absence of rapamycin, like the corresponding native proteins (Figure 3), produced the sigmoidal-shaped bands (Figure S5A–5D). In addition, the Trp fluorescence spectrum of refolded rFKBP22 or CTD^+^ also completely overlapped with that of the respective native protein in the presence of rapamycin (Figure S5E). Previously, we demonstrated the complete refolding of the denatured CTD^+^ and a recombinant FKBP22 [12]. The data together suggest that urea-induced denaturation of all rapamycin-bound proteins are reversible in nature. Our results, particularly the transition regions of the curve-shaped bands in the TUGE pictures (Figures S5 and 3), also indicate that the rate of unfolding/refolding of all proteins are not identical. The comparatively fading transition region of the rapamycin-saturated CTD^+^ suggests a slow equilibrium between the folded and unfolded forms of this protein. Similarly, rapamycin-bound rFKBP22 seems to unfold/refold relatively slowly as the transition of this protein spanned a broader interval of urea concentration, compared with rapamycin-unbound rFKBP22.

Binding of the cognate ligand to a protein usually enhances its stability considerably [23], [25], [43]. To examine whether rapamycin binding stabilizes rFKBP22 and CTD^+^, the monophasic curves (Figures 2A–B) were analyzed by a two-state model [41] due to their best fitting (data not shown). The values of resulting thermodynamic parameters (such as *C*_m_, *m*, Δ*G* ^W^, and ΔΔ*G*) are presented in Table 1. The *C*_m_ values from the monophasic curve-like protein bands in the TUGE pictures (Figures 3A–B) were also determined by a similar way. Analysis revealed that the *C*_m_ and Δ*G* ^W^ values of rFKBP22 and CTD^+^ in the presence of rapamycin were significantly higher than those calculated in the absence of rapamycin (all *p* values <0.05). Additional analyses showed that the ΔΔ*G* (free energy change between the rapamycin-bound and rapamycin-free protein) values from different assays vary from ∼1.25 to ∼2.9 kcal mol^−1^. The results together indicate that rapamycin binding enhances the stabilities of both rFKBP22 and CTD^+^ appreciably. Similar stabilization of rFKBP22 was noticed in the presence of FK506 too (Figure S6).

**Table 1:** Thermodynamic parameters of the urea-induced unfolding of proteins^a^.

Protein/Protein-Rap	Assay type	*C*_m_ (M)	*m* (kcal mol^−1^ M^−1^)	Δ*G* ^W^ (kcal mol^−1^)	ΔΔ*G* (kcal mol^−1^)
rFKBP22	Far-UV CD	3.68±0.06	2.08±0.04	7.67±0.29	
rFKBP22	Trp fluorescence	3.54±0.01	1.72±0.05	6.10±0.22	
rFKBP22	TUGE	3.95±0.07	ND	ND	
rFKBP22-Rap	Far-UV CD	4.43±0.12	1.30±0.06	5.76±0.09	1.26±0.10
rFKBP22-Rap	Trp fluorescence	5.05±0.01	1.98±0.01	10.00±0.06	2.79±0.02
rFKBP22-Rap	TUGE	4.98±0.15	ND	ND	
CTD^+^	Trp fluorescence	3.25±0.05	1.14±0.17	3.72±0.64	
CTD^+^	TUGE	4.05±0.07	ND	ND	
CTD^+^-Rap	Trp fluorescence	5.72±0.07	1.20±0.08	6.84±0.37	2.87±0.16
CTD^+^-Rap	TUGE	4.95±0.21	ND	ND	

a Thermodynamic parameters of the urea-induced unfolding curves were determined as described in Materials and methods. Rap and ND denote rapamycin and not determined, respectively.

The classical unfolding analyses most times cannot identify those intermediates, which are less populated and possess short life span [44]–[46]. Besides, the unfolding curves, generated from the different spectroscopic data, would not coincide if an intermediate is formed during the denaturation of a protein. To determine whether urea-induced unfolding of CTD^+^ and rFKBP22 (bound/unbound with rapamycin) occurs with the formation of intermediates, the fraction of denatured protein molecules, determined from all the spectroscopic data (except the CD data of rapamycin-CTD^+^), were plotted against the related urea concentrations. All of the resulting curves were monophasic in nature (Figure 2C). The generated rFKBP22- or CTD^+^-specific denaturation curves (created as above) mostly superimposed as before [12], [17], confirming that drug-unbound proteins unfolded by a two-state mechanism at 0-7 M urea. In contrast, the monophasic curves specific to rapamycin-bound rFKBP22 did not coincide, indicating the formation of intermediate during the urea-induced unfolding of the drug-equilibrated rFKBP22.

To locate whether urea-induced denaturation of rapamycin-saturated proteins (rFKBP22 and CTD^+^) are indeed involved with the formation of any intermediate, we constructed phase diagrams [17], [47]–[49] by plotting the Trp fluorescence intensity values of these complexes at 320 nm against their Trp fluorescence intensities at 365 nm. All drug-protein complexes yielded a non-linear plot (Figure 2D and Inset) at different urea concentrations. While a zigzag plot was resulted by rapamycin-saturated CTD^+^ at ∼0–2.5 M urea, the same was produced by rapamycin-equilibrated rFKBP22 at ∼0–4 M urea. The data indicate the formation of some intermediates during the urea-induced unfolding of rapamycin-bound proteins. Synthesis of intermediate was partly supported by our TUGE data, which showed that refolding of rapamycin-bound rFKBP22 is most possibly not complete at 0–2 M and 6–8 M urea (Figure S5B). Currently, structure, function, and stability of the intermediate formed by rapamycin-bound CTD^+^/rFKBP22 are not known clearly.

### Thermal unfolding of the rapamycin-bound/unbound proteins

**Figure 4:**
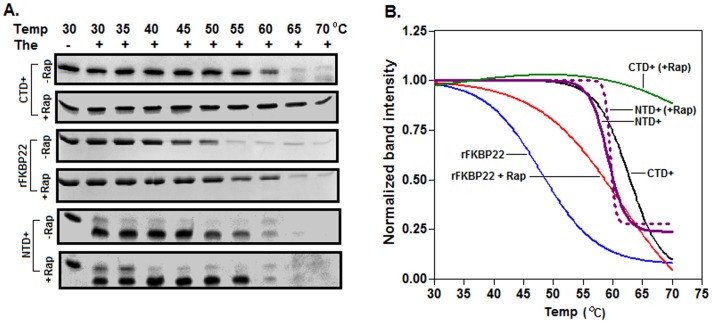
Limited proteolysis of the proteins with thermolysin at 30°–70°C. (A) SDS-13.5% PAGE analyses of the indicated proteins digested with thermolysin (The). (B) The intensities of the protein (undigested) bands were determined (by scanning the gel pictures in Panel A with a Gel Doc system), normalized with respect to that of the same at 30°C, and plotted versus the corresponding temperatures.

To understand whether rapamycin binding enhances the thermal stabilities of rFKBP22 and CTD^+^ too, we investigated the thermal unfolding of these proteins (pre-equilibrated with or without rapamycin) using various probes like limited proteolysis (with thermolysin), CD spectroscopy and Trp fluorescence spectroscopy. Figure 4A showed that thermolysin-mediated cleavage of rFKBP22 or CTD^+^ was started at relatively higher temperature in the presence of rapamycin. In contrast, NTD^+^ that does not bind rapamycin [12] was cleaved equally by thermolysin in the presence and absence of rapamycin. As thermolysin activity was not arrested by rapamycin at 30°–70°C, the cleavage of rapamycin-equilibrated rFKBP22/CTD^+^ by thermolysin at relatively higher temperatures might be owing to the decreased denaturation of these proteins in the presence of this PPIase inhibitor. To acquire comprehensive information about the stabilities of rFKBP22 and CTD^+^ (pre-equilibrated with or without rapamycin), *T*_m_ (the temperature at which 50% of the protein was digested by thermolysin) values were determined by plotting the band intensities of all undigested proteins (extracted from Figure 4A) against the respective temperatures. The resulting curves demonstrated that the *T*_m_ values for the rFKBP22- and CTD^+^-rapamycin complexes are ∼61.25° and ∼84.08°C, whereas, those for rFKBP22 and CTD^+^ are ∼48° and ∼62.71°C, respectively (Figure 4B). In contrast, *T*_m_ value for NTD^+^ in the presence or absence of rapamycin was found to be ∼59°C. The data together indicated that rapamycin binding enhanced the thermal stabilities of rFKBP22 and CTD^+^ appreciably.

**Figure 5:**
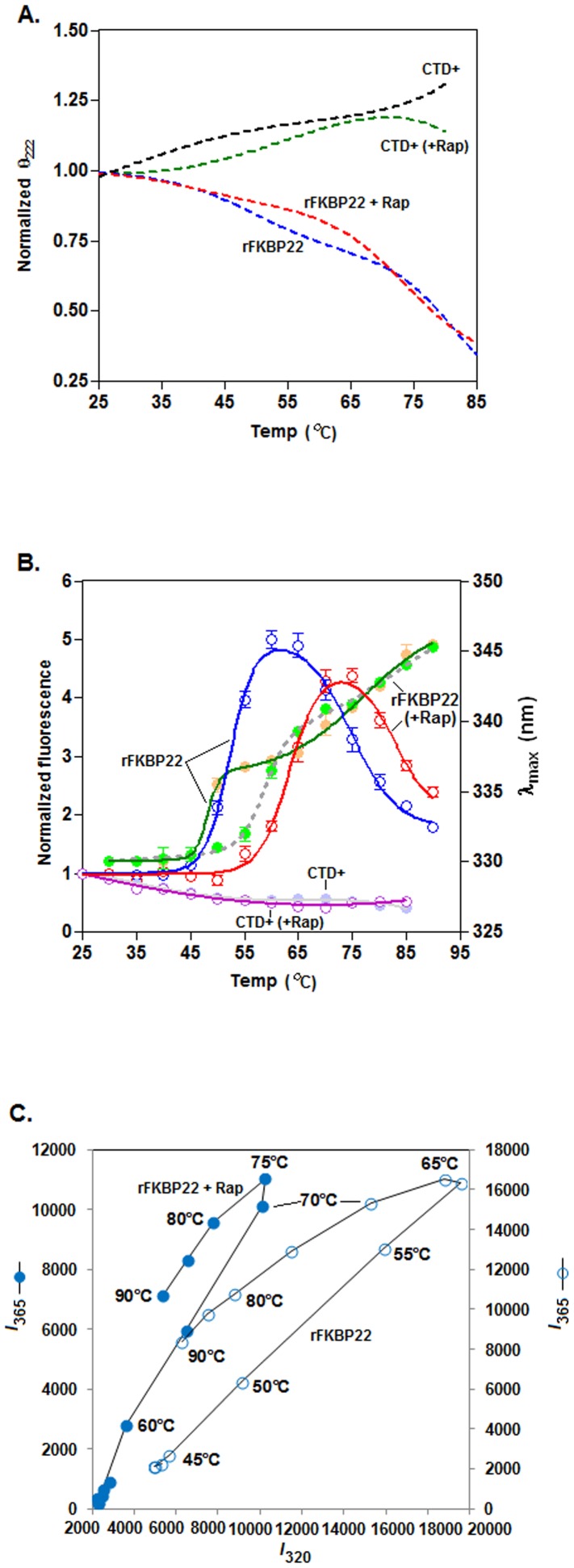
Thermal unfolding of of rFKBP22 and CTD^+^ in the absence and presence of rapamycin. (A) The θ_222_ (ellipticity at 222 nm) values, obtained from the CD spectra of the indicated proteins (saturated with or without Rap) at 25°–85°/95°C (Figures S7A–7D), were normalized with respect to that of the same at 25°C. The resulting normalized θ_222_ values were plotted versus the related temperatures. All lines through the CD values are the best-fit curves. (B) The intrinsic Trp fluorescence intensity values at 330 nm (for rFKBP22 bound with or without rapamycin) and at 347 nm (for CTD^+^ saturated with or without rapamycin) were similarly calculated from the Trp fluorescence spectra (Figures S7E–7H), normalized and plotted against the corresponding temperatures. The *λ*_max_ values associated with the rapamycin-equilibrated rFKBP22 and rFKBP22 at 25°–90°C were extracted too from the Trp fluorescence spectra (mentioned above) and plotted against the relevant temperatures. All lines through the fluorescence intensity or *λ*_max_ values are the best-fit curves. (C) Phase diagrams showing the unfolding of the indicated proteins at 25°–90°C. *I*_320_ and *I*_365_ denote the fluorescence intensity values (derived from Figures S7E–7H) of a protein at 320 nm and at 365 nm, respectively.

The negative CD values of rapamycin-bound rFKBP22 or rFKBP22 at 222 nm, derived from Figure S7, were gradually reduced, whereas those of rapamycin-equilibrated CTD^+^ or CTD^+^ at 222 nm were slowly increased upon raising the temperature from 25° to 85°C (Figure 5A). For reasons not known, curves specific to rapamycin-bound or rapamycin-unbound rFKBP22/CTD^+^ could not indicate clearly about the mechanism of thermal unfolding or the stability of these macromolecules. The rapamycin-bound rFKBP22 and rFKBP22, however, yielded biphasic curves at 25°–90°C (Figure 5B) when the Trp fluorescence intensities of these proteins at 330 nm or the associated *λ*_max_ values were plotted against the corresponding temperatures. Both the fluorescence intensities and the associated *λ*_max_ values of rapamycin-bound rFKBP22 and rFKBP22 were started increasing dramatically when their incubation temperatures were raised from ∼55° to 70°C and from ∼40° to 60°C, respectively. Such increase of fluorescence intensity at the beginning of unfolding could be due to the quenching fluorescence in the native rFKBP22. Many proteins (such as human FKBP, human methionyl-granulocyte stimulating factor, etc.) showed quenching of Trp fluorescence at the folded form [50]–[52]. At 70°–75°C, the fluorescence intensities and the *λ*_max_ values of the rFKBP22-rapamycin complex remained, however, mostly unaltered, whereas, those of rFKBP22 was nearly static at 60°–65°C. Thereafter, Trp fluorescence intensities of the drug-bound and drug-free proteins were decreased gradually with increasing temperatures and finally, tended to saturate at 85°C and higher temperatures. Conversely, the coupled *λ*_max_ values were increased gradually to 345 nm. Interestingly, CTD^+^ (bound or unbound with rapamycin) produced curves with no such transition when the equivalent Trp fluorescence spectroscopic data were plotted by similar manner (Figure 5B). The phase diagrams, created by plotting the Trp fluorescence intensities of rFKBP22 (bound/unbound with rapamycin) at 320 nm versus its Trp fluorescence intensities at 365 nm, showed that this protein in the absence and presence of rapamycin yielded non-linear plots with the notches at ∼60° and ∼75°C, respectively (Figure 5C). Taken together, we suggest that temperature-induced unfolding of the rapamycin-bound rFKBP22 and rFKBP22 occur by a three-state mechanism [42], [43] with the production of a stable intermediate at ∼60° and ∼75°C, respectively. In addition, thermal unfolding of rapamycin-equilibrated rFKBP22 initiated at temperature, which is higher than that of rFKBP22. On the contrary, there was possibly little unfolding of the drug-bound CTD^+^ and CTD^+^ at 25°–85°C.

**Figure 6:**
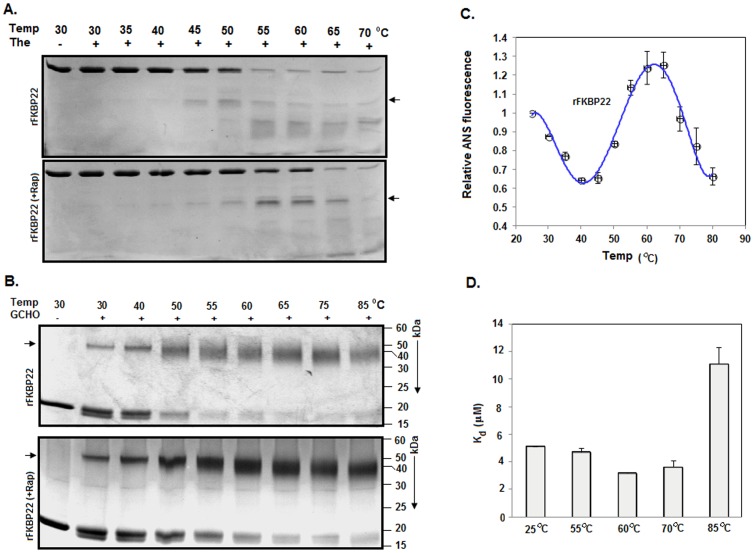
Characterization of the intermediates. (A) SDS-13.5% PAGE analysis of the fragments obtained from partial proteolysis of rFKBP22 (attached with or without rapamycin) with thermolysin (The) at 30°–70°C. Arrow indicates the additional protein fragments produced during proteolysis. (B) Chemical cross-linking. Proteins generated from glutaraldehyde (GCHO)-mediated cross-linking of rFKBP22 (attached with or without rFKBP22) at 30°–70°C were analyzed by SDS-13.5% PAGE. Arrow indicates dimeric rFKBP22 (attached with or without rapamycin). (C) ANS fluorescence of rFKBP22 at different temperatures. (D) Determination of *K*_d_ values for rapamycin-rFKBP22 interactions at different temperatures.

To obtain clues about the formation of the intermediates, we performed partial proteolysis of these proteins with thermolysin at 30°–70°C. Analyses of the proteolytic fragments from the rapamycin-equilibrated rFKBP22 and rFKBP22 revealed the generation of an additional protein fragment (with the molecular mass of ∼15 kDa) mostly at ∼55°–65°C and 45°–55°C, respectively (Figure 6A), indicating the synthesis of intermediates during their thermal unfolding. It is not understood why proteolysis showed the formation of intermediates at relatively lower temperatures. Both the intermediates were, however, found to retain sufficient extents of both secondary and tertiary structures (Figures 5A and 5B). A chemical crosslinking study of rFKBP22 (bound/unbound with rapamycin) at 30°–85°C revealed that the intermediates remained as the dimers in aqueous solution (Figure 6B). The apparent increase of the amount of dimeric intermediate at 50°C and higher temperatures might be due to the exposure of additional Lys residues (such as Lys86) besides Lys49. The ANS fluorescence intensity of rFKBP22 was increased to a maximum level at 60°C (Figure 6C), suggesting that rFKBP22 intermediate is composed of higher hydrophobic surface area than the native rFKBP22. Interestingly, ANS fluorescence intensity of rKBP22 was decreased partly (possibly due to thermal quenching) when its incubation temperature was raised from 30° to 40°C. A similar study with the rapamycin-bound rFKBP22 failed, as it showed aggregation in the presence of ANS at 60°C and higher temperatures. The *K*_d_ value for the interaction between rFKBP22 and rapamycin at 60°C was found to be significantly less than that determined for the same at 25°C (*p* = 0.012), indicating that rFKBP22 intermediate possesses a relatively higher rapamycin binding affinity than the native rFKBP22 (Figure 6D). At other higher temperatures, rapamycin binding affinity of rFKBP22 is either less or similar to that at 25°C.

To determine whether thermal unfolding of the rapamycin-bound rFKBP22 and rFKBP22 are reversible in nature, we investigated different properties of their probable refolded forms using different probes. Limited proteolysis with trypsin and subsequent analysis (Figure S8A) showed that the masses of all proteolytic fragments from the probable refolded rFKBP22 (bound/unbound to rapamycin) were almost identical to those produced by similar digestion of the native protein (also bound/unbound to rapamycin), indicating the restoration of two domains of rFKBP22 by proper refolding. The CD spectrum (Figure S8B) and the Trp fluorescence spectrum (Figure S8C) of refolded rFKBP22 (bound or unbound with rapamycin) were mostly superimposed with those of respective native proteins. The *λ*_max_ value of refolded rFKBP22 also matched with that of native rFKBP22 in the presence and absence of rapamycin. RNase T1 refolding assay [12] revealed that both refolded and native rFKBP22 folded the denatured RNase T1 by nearly identical manner (Figure S8D). The refolded rFKBP22, therefore, had PPIase activity like that of native rFKBP22. Taken together, we suggest that thermal unfolding of rFKBP22 (equilibrated with or without rapamycin) is completely reversible in nature.

To understand the rapamycin-mediated stabilization of rFKBP22 accurately, the values of different thermodynamic parameters were determined from the biphasic thermal unfolding curves (Figure 5B) and listed in Table 2. Of the parameters, the *T*_m_ values linked to the unfolding transitions of rapamycin-equilibrated rFKBP22 were significantly higher than the corresponding *T*_m_ values of rFKBP22 (all *p* values <0.05). The ΔΔ*G*1 (the difference of free-energy change between the rapamycin-bound protein and rapamycin-unbound protein for folded to intermediate state transition) and ΔΔ*G*2 (the difference of free-energy change between the rapamycin-bound protein and rapamycin-unbound protein for intermediate to unfolded state transition) values were determined (using *T*_m_ and other thermodynamic parameters in Table 2) and found to be quite high too. The values of enthalpy change, determined for rFKBP22 or rapamycin-bound rFKBP22, are relatively small. Similar values of enthalpy change were, however, reported for apoflavodoxin that carries 168 residues [42]. The *T*_m_ and ΔΔ*G* values, however, suggested the stabilization of rFKBP22 by rapamycin.

**Table 2:** Thermodynamic parameters for the heat-induced unfolding of proteins^a^.

Protein/Protein-Rap	*T*_m1_ (°C)	*T*_m2_ (°C)	Δ*H*_m1_ (kcal mol^−1^)	Δ*H*_m2_ (kcal mol^−1^)	ΔΔ*G*1 (kcal mol^−1^)	ΔΔ*G*2 (kcal mol^−1^)
rFKBP22	51.67±0.44	75.41±0.71	16.01±0.35	15.62±0.07		
rFKBP22-Rap	64.39±0.78	82.34±0.08	22.92±0.55	19.56±0.05	3.94±0.12	1.42±0.01

a Thermodynamic parameters of the thermal unfolding curves were determined as described in Materials and methods. Rap indicates rapamycin.

Our studies indicated the synthesis of intermediates during the thermal and urea-induced unfolding of rapamycin-bound rFKBP22. Thermal unfolding of rFKBP22 also occurred via the formation of intermediate. In contrast, our previous [12], [17] and present studies (particularly, spectroscopic data) could not clearly demonstrate the production of intermediate when rFKBP22 was denatured by urea. Formation of intermediate during the unfolding of rFKBP22 (bound with/without rapamycin) is, however, not unexpected as this macromolecule carries two domains and is dimeric in solution. Unfolding intermediate, generated by FKBP22, was also reported from the homologous *Shewanella* FKBP22 [16]. The putative intermediate synthesized from *Shewanella* FKBP22 had not been characterized yet. Unfolding of a trigger factor in the presence GdnCl occurred via a three-state mechanism [53]. Urea-induced denaturation of a mycobacterial PPIase followed a three-state mechanism in the presence and absence of the cognate drug [54]. Interestingly, thermal unfolding of this enzyme led to its aggregation at temperatures of >70°C [54]. The intermediate accumulated from His-FKBP22 (a recombinant *E. coli* FKBP22 slightly different from rFKBP22) in the presence of GdnCl was, however, suggested to be composed of different oligomeric forms of this macromolecule [12]. Like the intermediate produced from the GdnCl-induced unfolding pathway, intermediates generated from the thermal unfolding of rFKBP22 (bound with/without rapamycin) or from the urea-induced unfolding of rFKBP22-rapamycin complex may not be a true ‘molten globule’ as these are composed of sufficient extent of tertiary structure [43].

Human pathogens that produce Mip and the Mip-like proteins became resistant to the most antimicrobial agents available in the clinics today [55]–[58]. To eradicate such resistant microorganisms, new drugs are needed to be screened or developed urgently. A new drug against a target protein (say, Mip) could be screened with ease if binding of the putative drug candidate enhances the stability (or midpoint of unfolding transition) of this macromolecule substantially [28], [33], [59]. Previously, a tool, which determined the stability and the rapamycin binding affinity of human FKBP12 efficiently, was suggested to be useful for screening new drugs against this target [28]. Our data for the first time showed that there are stabilization of a Mip-like protein and its C-terminal derivative in the presence of the cognate drugs. The results suggest that an assay system (as mentioned above) could also be developed using rFKBP22 or CTD^+^ for screening the rapamycin-like inhibitors in the future.

## Conclusions

*E. coli* FKBP22 and the related PPIase enzymes bind rapamycin and FK506 using their C-terminal domains. The present investigation revealed that the tertiary structures of rFKBP22 and CTD^+^ were slightly changed upon binding to rapamycin. Urea-induced unfolding of all rapamycin-bound proteins occurred via the synthesis of intermediates. Temperature-induced unfolding of rFKBP22 or rFKBP22-rapamycin complex also generated intermediates. Interestingly, CTD^+^ or rapamycin-bound CTD^+^ was very little unfolded at the temperatures those unfolded rFKBP22 (saturated with or without rapamycin) substantially. Further studies indicated that all intermediates generated in the thermal unfolding pathway exist as the dimers in solution, and are composed of adequate extents of tertiary and secondary structures. Intermediate produced from rFKBP22 also contained enhanced hydrophobic surface and possessed substantial rapamycin binding ability. Denaturation with either unfolding agent was, however, reversible in nature. Unfolding experiments also demonstrated that rapamycin binding stabilized both the proteins significantly. Possible implications of the stability data in the drug screening have been discussed.

## Supporting Information

Figure S1 **Development and visualization of the model structures.** Three-dimensional model structures of the CTD^+^-rapamycin complex (A) and CTD^+^ (B) were generated as described in Materials and methods. The ribbon, arrow, and tube indicate α-helix, β-sheet and loop, respectively. Rap indicates rapamycin. (C) Superimposition of the α-carbon backbone of CTD^+^-Rap complex (Blue) on the similar backbone of CTD^+^(red).
(TIF)

Figure S2 **Intrinsic Trp fluorescence spectra of proteins.** The intrinsic Trp fluorescence spectra of the indicated proteins (saturated/unsaturated with FK506) were recorded at 25°C.
(TIF)

Figure S3 **Alignment of the Mip-like proteins.** The indicated Mip-like proteins were aligned by ClustalW program.
(TIF)

Figure S4 **Urea-induced unfolding of the rapamycin-bound/unbound rFKBP22 and CTD^+^.** The far-UV CD (A-D) and intrinsic Trp fluorescence spectra (E–H) of the indicated proteins in the presence of 0–7 M urea are shown.
(TIF)

Figure S5 **Refolding of the urea-treated proteins.** The indicated proteins (pre-equilibrated with/without rapamycin) were denatured with 7 M urea followed by their analysis using the transverse urea gradient polyacrylamide gel electrophoresis (A–D). The denatured proteins were refolded as described in Materials and methods. The intrinsic Trp fluorescence spectra of the denoted native, refolded, and denatured protein are shown in panel E.
(TIF)

Figure S6 **Unfolding of the FK506-bound/unbound rFKBP22.** Samples containing rFKBP22 (pre-saturated with/without 4 molar excess of FK506) were exposed to 0–7 M urea followed by the recording of their intrinsic Trp fluorescence spectra by a standard method. The Trp fluorescence intensity values were extracted (from the spectra), normalized and plotted against the corresponding urea concentrations.
(TIF)

Figure S7 **Temperature-induced unfolding of the rapamycin-bound/unbound rFKBP22 and CTD^+^.** The far-UV CD (A–D) and intrinsic Trp fluorescence spectra (E–H) of the indicated proteins at 25°–95°C are presented.
(TIF)

Figure S8 **Refolding of the heat-treated rFKBP22.** Heat-exposed rFKBP22 (pre-equilibrated with/without rapamycin) was refolded followed by its analysis using trypsinolysis (A), far-UV CD spectroscopy (B), intrinsic Trp fluorescence spectroscopy (C), and RNase T1 refolding assay (D). The native and denatured rFKBP22 were used in the study as controls.
(TIF)
