# Supplementary figures and images for: Inhibitor-Induced Conformational Stabilization and Structural Alteration of a Mip-Like Peptidyl Prolyl cis-trans Isomerase and Its C-Terminal Domain
Source: PLoS One. 2014 Jul 29;9(7):e102891. doi: 10.1371/journal.pone.0102891 (PMC4114562; doi:10.1371/journal.pone.0102891)

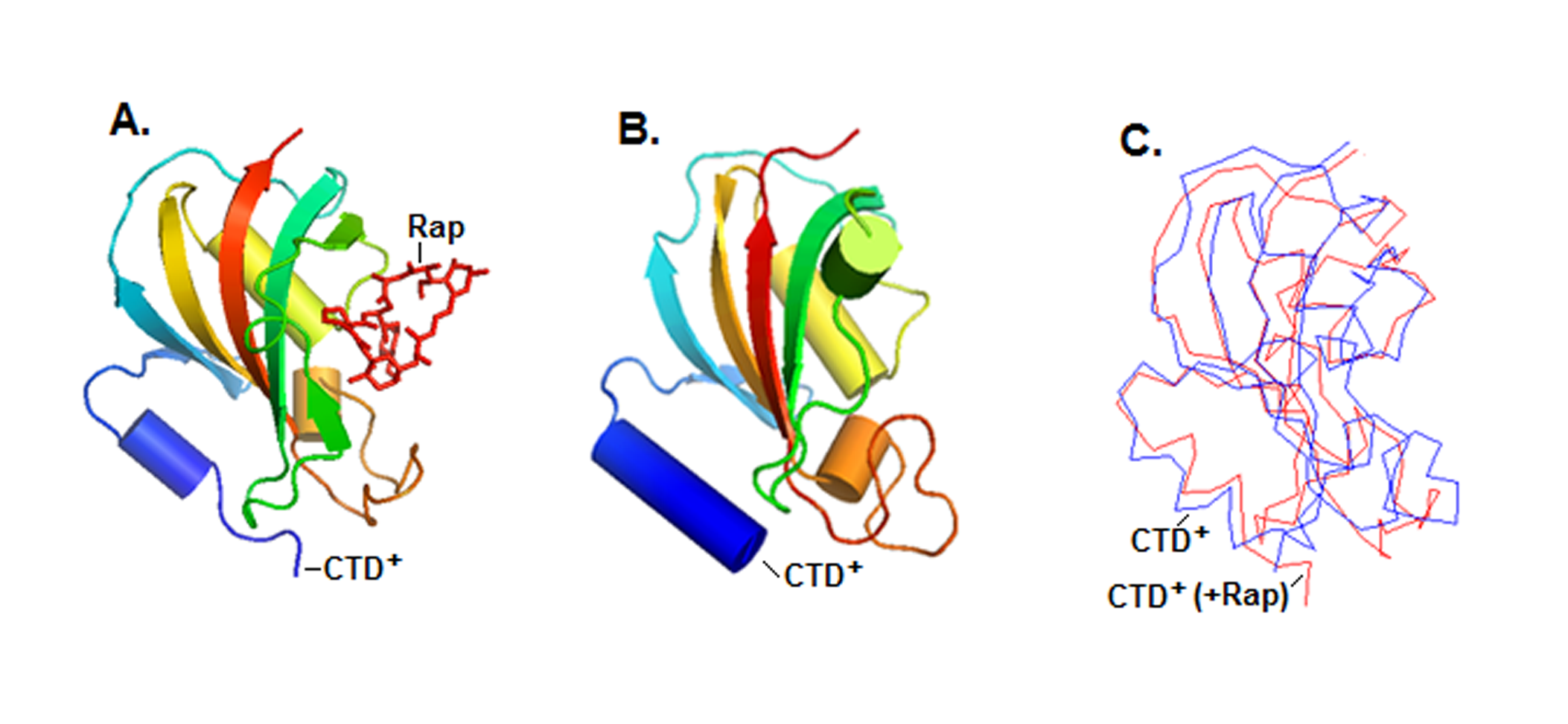

Supplement: Figure S1 — Development and visualization of the model structures. Three-dimensional model structures of the CTD+-rapamycin complex (A) and CTD+ (B) were generated as described in Materials and methods. The ribbon, arrow, and tube indicate α-helix, β-sheet and loop, respectively. Rap indicates rapamycin. (C) Superimposition of the α-carbon backbone of CTD+-Rap complex (Blue) on the similar backbone of CTD+(red). (TIF) [file pone.0102891.s001.tif]

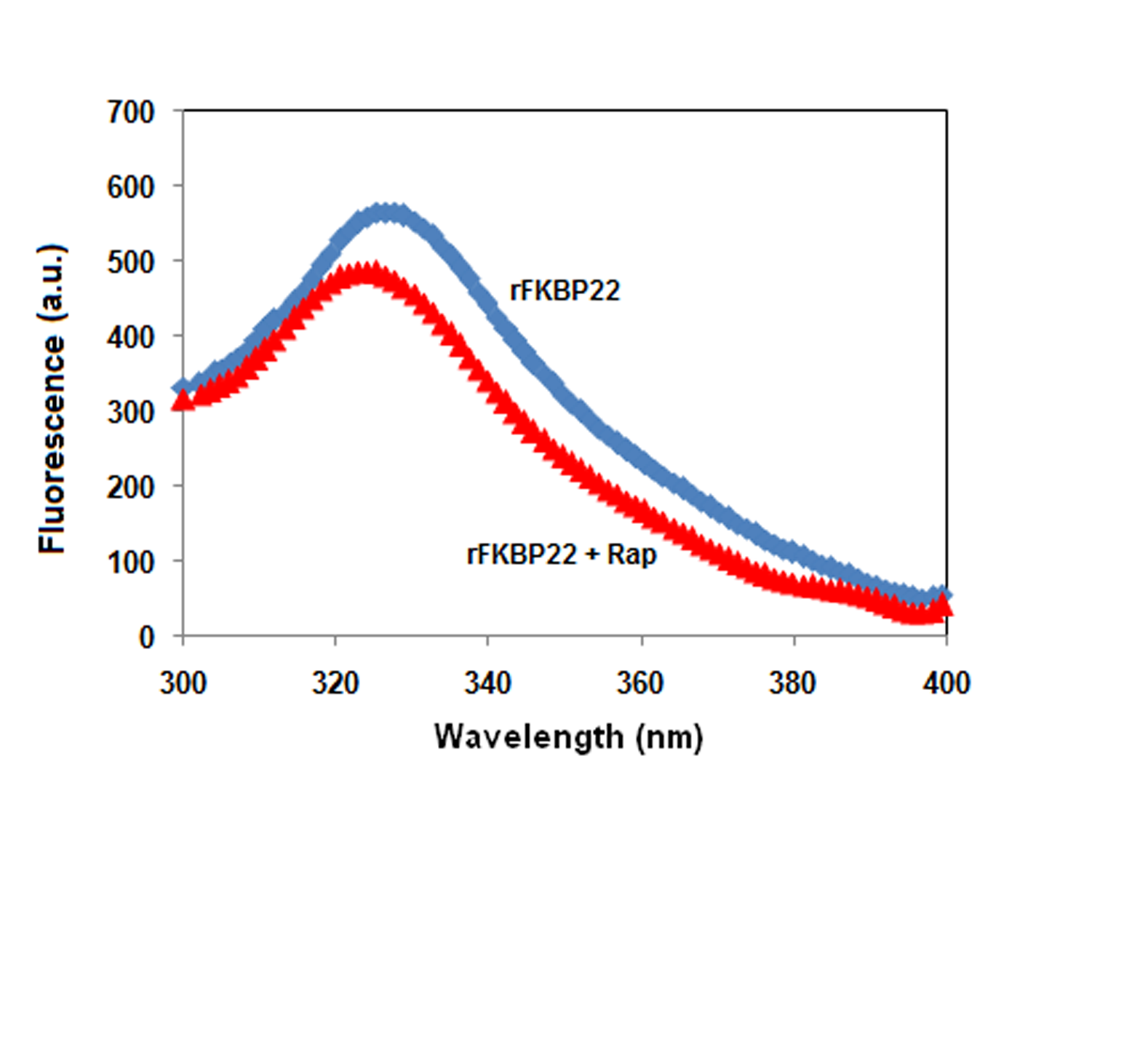

Supplement: Figure S2 — Intrinsic Trp fluorescence spectra of proteins. The intrinsic Trp fluorescence spectra of the indicated proteins (saturated/unsaturated with FK506) were recorded at 25°C. (TIF) [file pone.0102891.s002.tif]

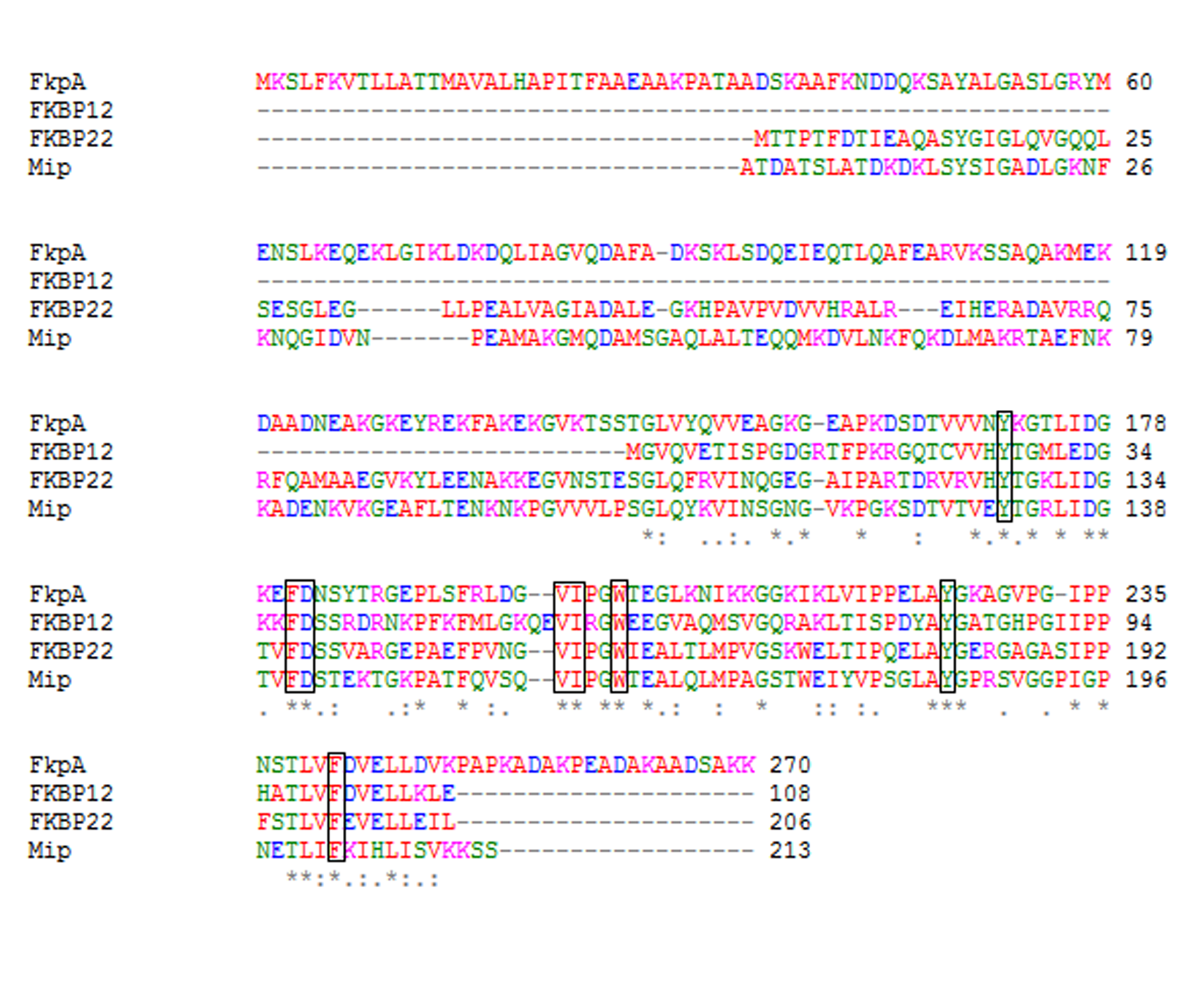

Supplement: Figure S3 — Alignment of the Mip-like proteins. The indicated Mip-like proteins were aligned by ClustalW program. (TIF) [file pone.0102891.s003.tif]

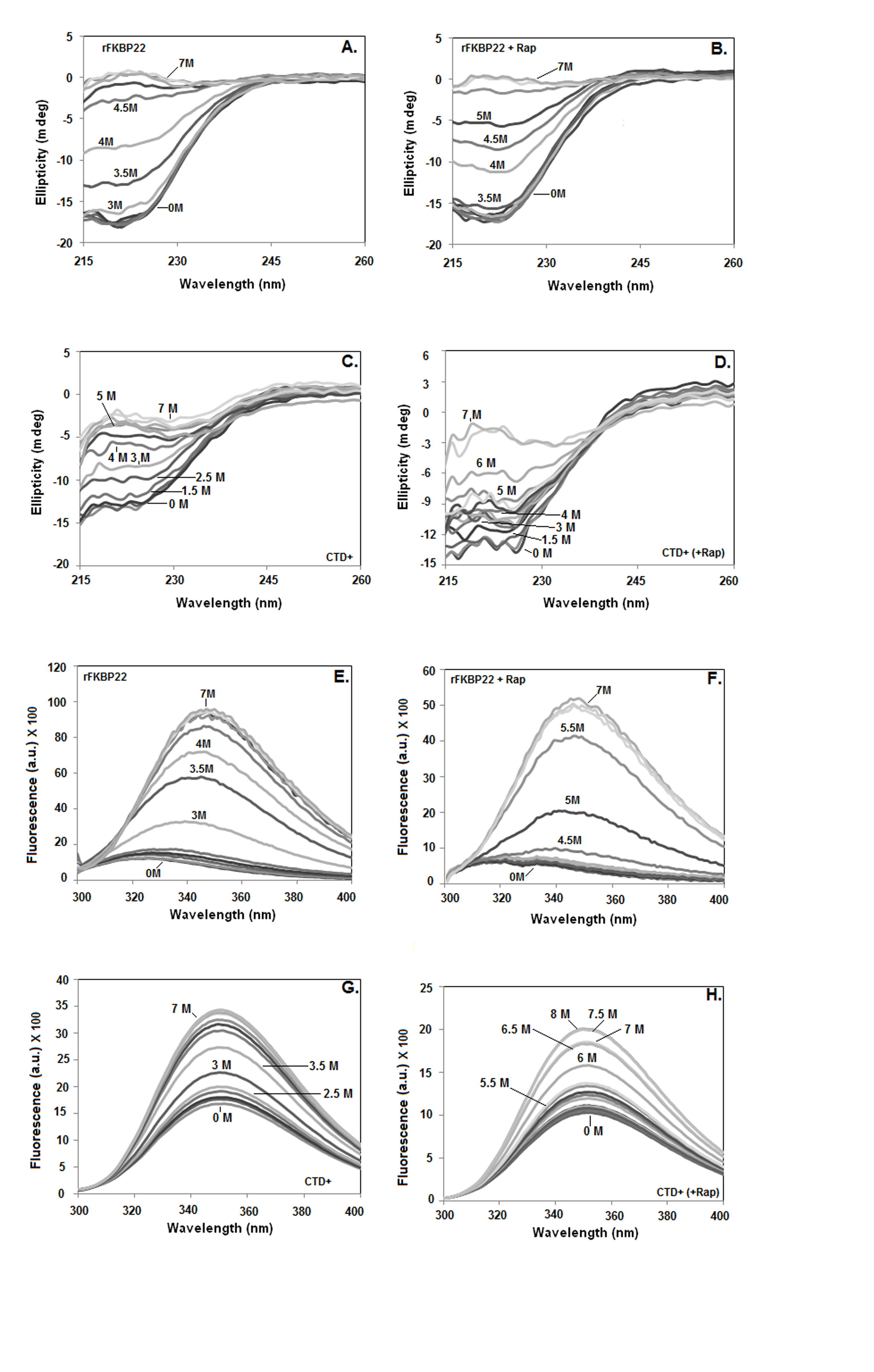

Supplement: Figure S4 — Urea-induced unfolding of the rapamycin-bound/unbound rFKBP22 and CTD+. The far-UV CD (A-D) and intrinsic Trp fluorescence spectra (E–H) of the indicated proteins in the presence of 0–7 M urea are shown. (TIF) [file pone.0102891.s004.tif]

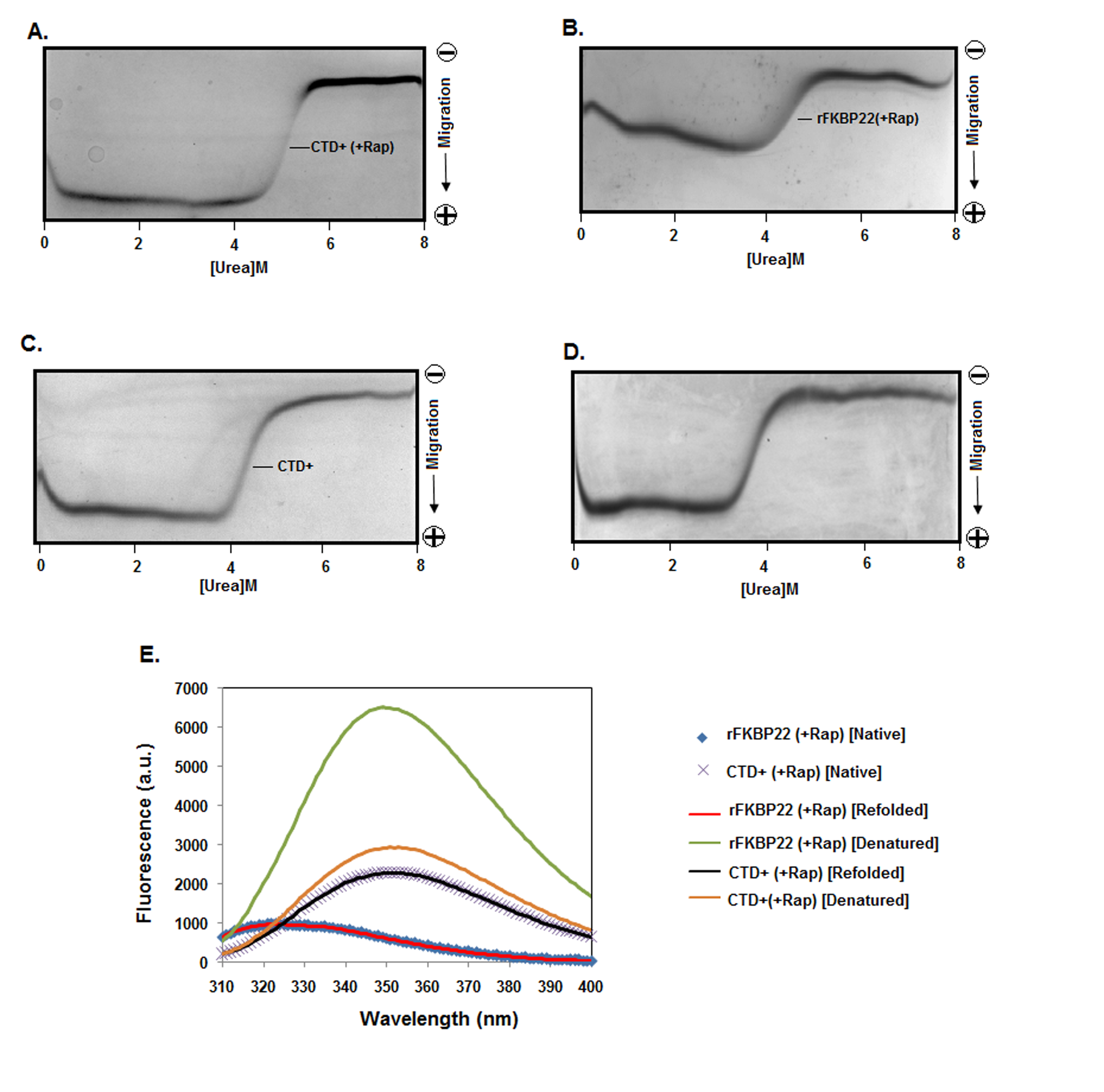

Supplement: Figure S5 — Refolding of the urea-treated proteins. The indicated proteins (pre-equilibrated with/without rapamycin) were denatured with 7 M urea followed by their analysis using the transverse urea gradient polyacrylamide gel electrophoresis (A–D). The denatured proteins were refolded as described in Materials and methods. The intrinsic Trp fluorescence spectra of the denoted native, refolded, and denatured protein are shown in panel E. (TIF) [file pone.0102891.s005.tif]

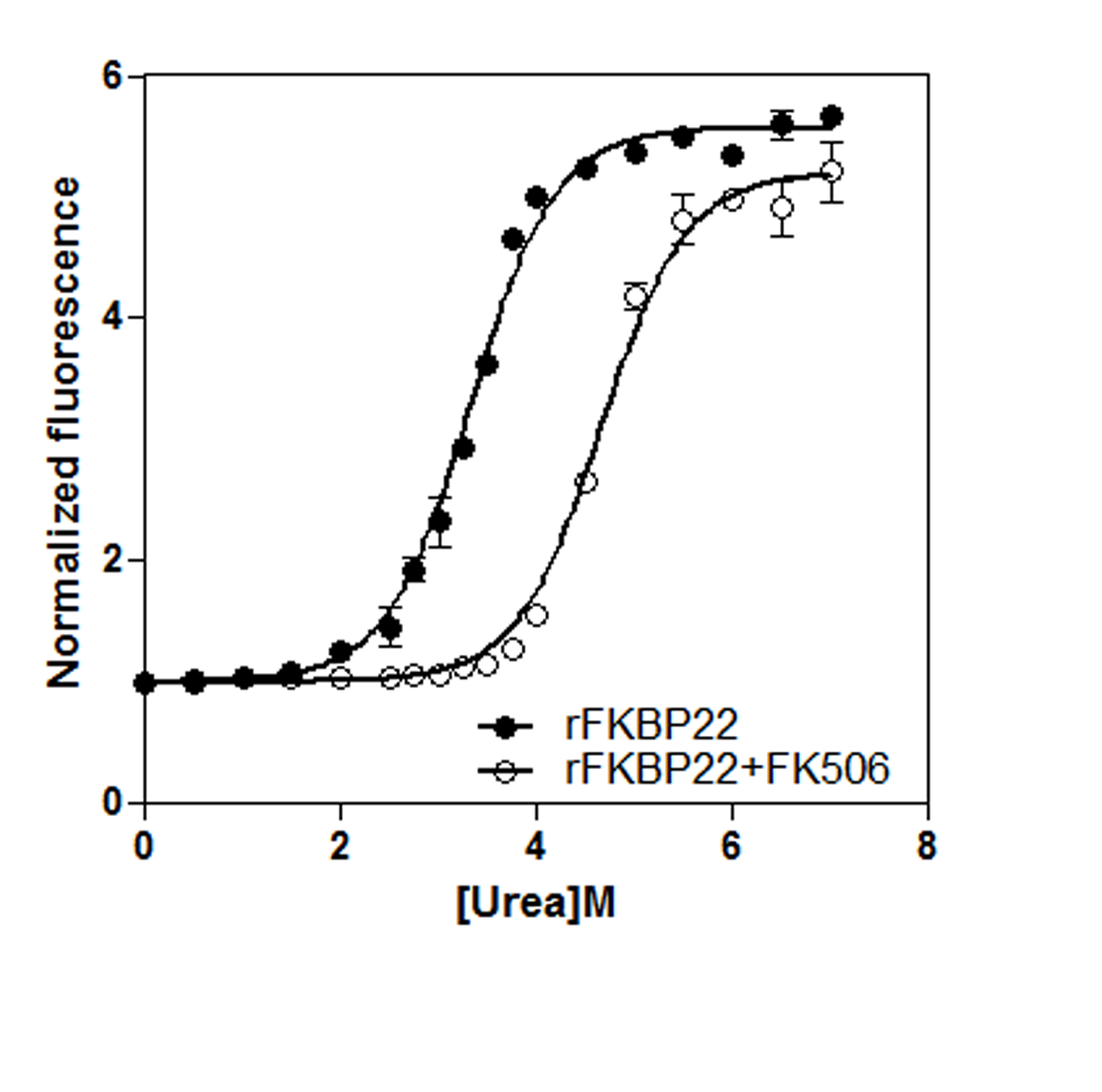

Supplement: Figure S6 — Unfolding of the FK506-bound/unbound rFKBP22. Samples containing rFKBP22 (pre-saturated with/without 4 molar excess of FK506) were exposed to 0–7 M urea followed by the recording of their intrinsic Trp fluorescence spectra by a standard method. The Trp fluorescence intensity values were extracted (from the spectra), normalized and plotted against the corresponding urea concentrations. (TIF) [file pone.0102891.s006.tif]

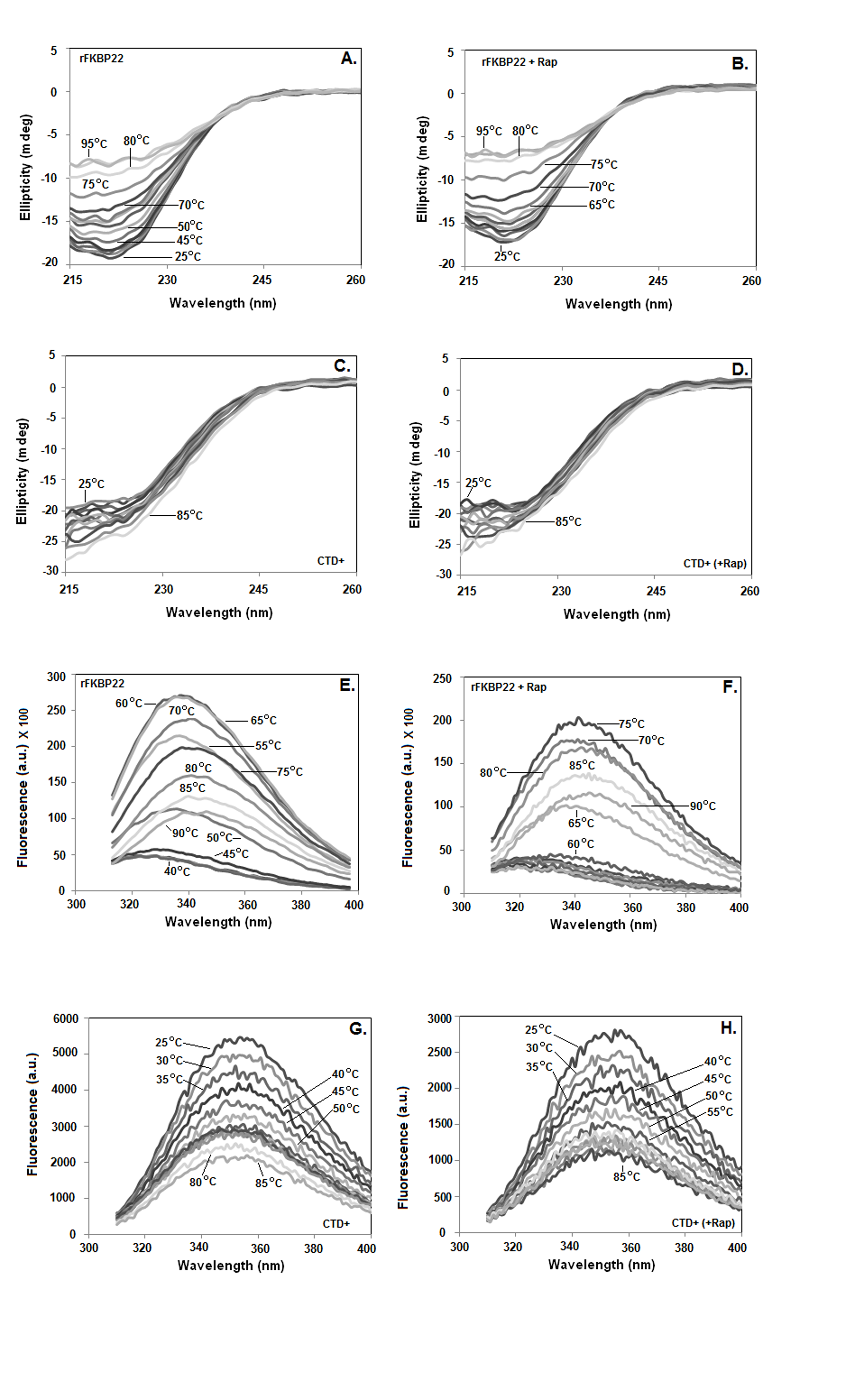

Supplement: Figure S7 — Temperature-induced unfolding of the rapamycin-bound/unbound rFKBP22 and CTD+. The far-UV CD (A–D) and intrinsic Trp fluorescence spectra (E–H) of the indicated proteins at 25°–95°C are presented. (TIF) [file pone.0102891.s007.tif]

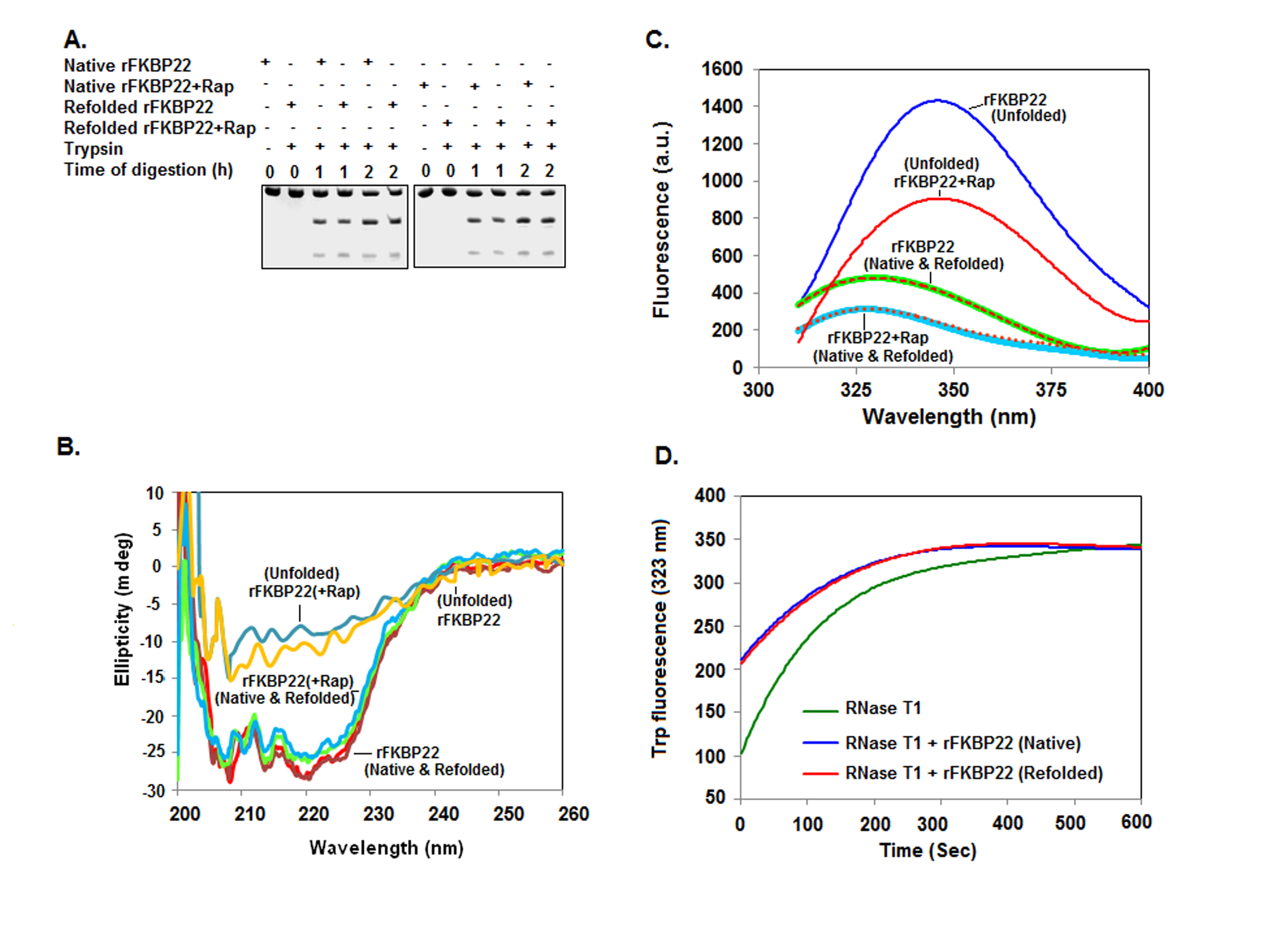

Supplement: Figure S8 — Refolding of the heat-treated rFKBP22. Heat-exposed rFKBP22 (pre-equilibrated with/without rapamycin) was refolded followed by its analysis using trypsinolysis (A), far-UV CD spectroscopy (B), intrinsic Trp fluorescence spectroscopy (C), and RNase T1 refolding assay (D). The native and denatured rFKBP22 were used in the study as controls. (TIF) [file pone.0102891.s008.tif]
